# 30 years revisit survey for long-term changes in the Antarctic subtidal algal assemblage

**DOI:** 10.1038/s41598-020-65039-4

**Published:** 2020-05-21

**Authors:** Young Wook Ko, Han-Gu Choi, Dong Seok Lee, Jeong Ha Kim

**Affiliations:** 1 Division of Polar Life Sciences, Korea Polar Research Institute, Incheon, 21990 Republic of Korea; 2 Department of Biological Sciences, Sungkyunkwan University, Suwon, 16419 Republic of Korea

**Keywords:** Biodiversity, Community ecology

## Abstract

A long-term change of a subtidal macroalgal assemblage has been investigated in Maxwell Bay, King George Island (KGI) of the Antarctic coast by a revisit survey after 30 years. Field surveys were done by SCUBA diving at six sites in 2016–2018 to directly compare with the previous survey conducted in 1988–1993 at the same sites. The total number of macroalgal species was similar between the previous and the present survey, 25 and 27 species respectively. However, the macroalgal assemblage changed substantially with the average similarity of 48.2% between the two surveys. Also, the species-level abundance showed a high variability between surveys. On the other hand, over the 30 years interval there was little overall change at the between-site level hierarchical structure in the subtidal communities of Maxwell Bay. The sites near the penguin rookery consistently showed the highest biodiversity, indicating the importance of land-based nutrients input in Antarctic coastal habitats. A noticeable pattern change over 30 years was the increase of *Desmarestia* complex and *Plocamium cartilagineum* and the decrease of *Himantothallus grandifolius*. Both groups are still dominant, but the shift from *Himantothallus* to *Desmarestia-Plocamium* may reflects temperature rise on the Maxwell Bay coast compared to the past.

## Introduction

Man-induced global climate change is causing gradual changes in biological systems^1–4^. As warming continues, the area where life can be inhabited extends to the polar regions, high mountains, and deep seas, but at the same time ecological succession occur due to changes in biological niche, that is, the extinction and invasion of organisms^5,6^. It has been predicted that coastal benthic ecosystems^7^, marine benthic communities^8^, marine fauna^9^ and kelp forests^10^ will change gradually caused by global warming. Many studies on various responses of macroalgae to climate changes have been done in the temperate and tropical regions^11–15^, these studies focused on population fluctuations and assemblage changes of macroalgae related to changes in seawater temperature and acidification.

The polar region has warmed more than twice as much as the global average since 1850^16^, and the Antarctic Peninsula has the highest surface temperature rise in the Southern Hemisphere^17^. Despite this climatic significance in Antarctica, a few experimental studies have been recently reported which predict the fate of future populations from the algal demographic data such as mortality, growth and reproduction in relation to water temperature rise and pH drop^18,19^. Such studies, however, predicted assemblage changes on the base of population level fluctuations, thus direct observation of macroalgal assemblage level changes were still lacking. Recently, investigations on long-term assemblage change through the comparison with previously collected data, so called historical research, have been done in the Arctic region. For example, Kortsch, *et al*.^20^ reported changes, after 30 years, of macroalgal coverage and marine benthic assemblage and Bartsch, *et al*.^21^ compared seaweed biomass data with that of 16 years ago. Also, Filbee-Dextor *et al*.^10^ summarize the positive and negative effects of warming on kelp forests through kelp biomass information from 1973 to 2016. In the Antarctic, Mystikou, *et al*.^22^ compared seaweed diversity of the islands in the Antarctic Peninsula with the data collected 35 years ago. Due to the mismatch of the study sites between past and present, it is difficult to explain explicitly diversity change over time. However, our present study provides valuable information on seaweed species checklist that can be an useful reference for comparative study in the future.

**Figure 1:**
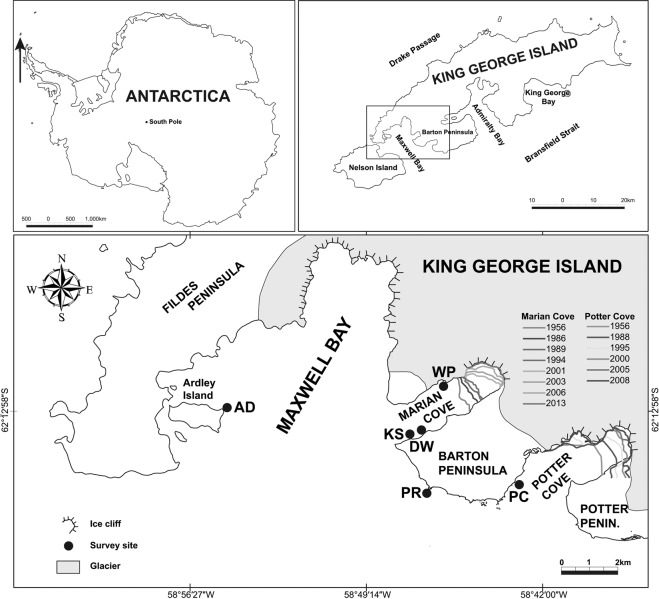
Map of the Maxwell Bay, King George Island, indicating the six sampling sites. Line marks represent the retreat of glacier since 1956, Marian Cove from Moon, *et al*.^8^ and Potter Cove from Rückamp, *et al*.^25^. The gray area denotes glacier cover.

South Shetlands Island (SSI) archipelago is located 120 km away from the north end of the Antarctic Peninsula and consists of many islands including King George Island (KGI). SSI is known for the highest biodiversity area in the Antarctic coast^23,24^, also related to the relatively low latitude compared to the rest of Antarctica. KGI is a region where warming is very visible. Since 1956, more than 1.7 km and 1.0 km of glaciers have retreated from Marian Cove and Potter Cove, respectively^8,25^ (Fig. 1). Also, since 1947, the warming trend of the annual mean atmospheric temperature showed 0.022 °C a^×1^, corresponding to an increase of about 1.1 °C over 49 years. The warming trend of air temperature during winter was 0.038 °C a^−1^, which was about two times stronger than in the other season^26^. King Sejong Station (KSS), located in KGI, was established in 1988. Macroalgal research was initiated at the beginning of KSS and focused on taxonomy and assemblage^27,28^. Chung and his colleagues investigated seaweed distribution and biomass at 17 intertidal and subtidal sites in Maxwell Bay, KGI, in 1988–1993, and compared assemblage between the sites. This pioneer research contains important reference data for a long-term monitoring of this macroalgal assemblage, but follow-up monitoring has not been carried out for the last decades.

The purpose of our study is to conduct a comparative study of the long-term changes at the same sites used in the past survey of about 30 years ago as well as to provide more detailed ecological data on current vertical and spatial distribution as baseline information for possible future monitoring. The present monitoring can also be another historical study in the Antarctic region, if such successive research continues, and will contribute to our understanding on community dynamics and ecosystem changes with relation to global warming and other various causes.

## Results

### Long-term change of algal assemblage

**Figure 2:**
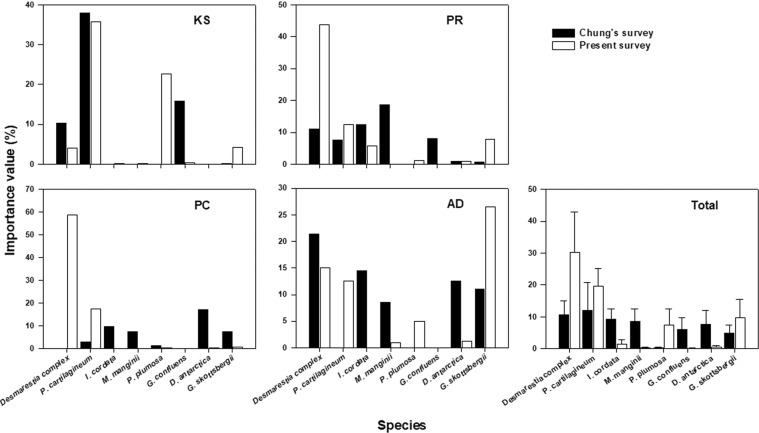
The importance value (IV) of major species between sampling times at four sites, in Maxwell Bay, King George Island; Bar represents mean IV; Eight major species with a SIMPER contribution of more than 5%.

**Figure 3:**
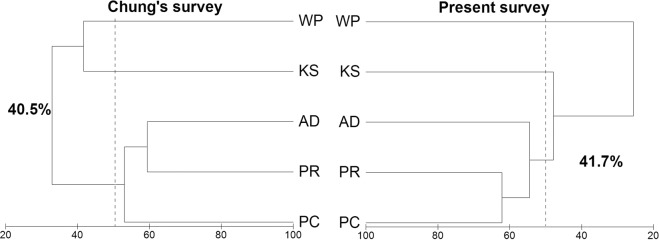
Cluster analysis of similarities of algal assemblage based on importance value between the five sites, at each sampling times, in Maxwell Bay, King George Island. Percentage number is similarity of overall site in each survey.

The species with notable quantitative increase were *Desmarestia* complex (mixed with *D. anceps* and *D. menziesii*: mainly composed of *D. menziesii)* and *Plocamium cartilagineum* in the past 30 years (Tables 1, 2). In the Chung’s 1988–93 survey (here-after Chung’s survey), an islet in the entrance of Potter Cove site (site PC) showed the highest diversity (Shannon-Wiener diversity index; H’, the parameters presented here are based on the abundance derived from the IV value; see Materials and Methods) at 2.59 (Table 1). In the present survey in 2016–18 (here-after present survey), an islet off Ardley Island site (site AD) showed the highest diversity of 2.08 and site PC showed relatively low at 1.38. It was noteworthy that the diversity of site PC was low due to dominance of *Desmarestia* complex (Table 2, Fig. 2). Despite a time difference of about 30 years, the macroalgal assemblage was little overall change in the hierarchical structure based on the similarity of assemblage between sites (Chung’s survey vs. present survey; Fig. 3). A closer look at the results of cluster analysis through similarity analysis, based on the similarity (Bray-Curtis similarity) of 40%, the Chung’s survey was divided into two groups of less disturbed site PR (an islet off penguin rookery site), PC, AD and disturbed site KS (pier of King Sejong Station site), WP (a southern shore of Weaver Peninsula site). Similarity of assemblage within sampling time was 40.5% in the Chung’s survey and 41.7% in the present survey (Fig. 3; Supplementary Table S1).

**Table 1:** Comparisons of benthic macroalgal composition and abundance in Maxwell Bay, King George Island between the Chung’s survey and the present survey; The data set was based on biomass from the Chung’s survey and coverage from the present survey; Exceptionally, the data set used at site PC in Chung’s survey was based on relative coverage.

	Chung’s survey					Present survey				
	KS	PR	PC	AD	WP	KS	PR	PC	AD	WP
**Chlorophyta**										
*Monostroma hariotii*			+	+	+	+		+		+
*Ulothrix australis*						+				
*Ulva bulbosa*			+							
**Chrysophyta**										
*Antarctosaccion applanatum*			+							
**Phaeophyta**										
*Petroderma maculiforme*							+			
*Adenocystis utricularis*			+							
*Halopteris obovata*									+	
*Desmarestia antarctica*		+	+++	++			+	+	+	
*Desmarestia* complex^a^	++	+++		+++		+	+++	+++	+++	
*Himantothallus grandifolius*	+	+++	+++	++	++	+	+	++	+++	
*Phaeurus antarcticus*		+		++				+	+	
*Cystosphaera jacquinotii*		+++	++				+			
*Ascoseira mirabilis*		+	++			+	+	+		
**Rhodophyta**										
*Antarcticothamnion polysporum*		+								
*Ballia callitricha*			+							
*Delisea pulchra*			+						+	
*Palmaria decipiens*	+	+	+	+	+	+	+		+	+++
*Callophyllis linguata*									+	
*Trematocarpus antarcticus*	+			+					+	
*Acanthococcus antarcticus*							+	+	+	+
*Gigartina skottsbergii*	+	+	++	+++	+	+	++	+	+++	
*Iridaea cordata*		+	++	+		+	+			
*Phyllophora ahnfeltioides*		+	+	+						
*Plocamium cartilagineum*	+	+	+	+		+++	++	+++	++	+
*Curdiea racovitzae*		+		+				+	++	
*Georgiella confluens*	+	+				+	+			
*Myriogramme manginii*		++	++	+		+		+	+	
*Neuroglossum ligulatum*							+			
*Pantoneura plocamioides*		+	+				+			
*Phycodrys antarctica*							+			
*Paraglossum lancifolium*							+			
*Picconiella plumosa*			+			++	+	+	+	
Unidentified species^b^		+		+				+	+	
Total species	7	15	18	13	4	12	17	14	16	4
Shannnon index	1.59	2.28	2.59	2.11	0.82	1.79	1.94	1.38	2.08	0.59

^a^Mixed with *D. anceps* and *D. menziesii* (mainly composed of *D. menziesii*).

^b^Not identifiable on the photograph.

^+^Biomass: <10 g wet wt. m^−2^; coverage: <5%; relative coverage: <10%.

^++^Biomass: >10 g wet wt. m^−2^ & <25 g wet wt. m^−2^; coverage: >5% & <10%; relative coverage: >10% & <15%.

^+++^Biomass: >25 g wet wt. m^-2^; coverage: >10%; relative coverage: >15%.

**Table 2:** Macroalgal assemblage state, species contributing (%) of the Chung’s survey and the present survey, and dissimilarity of macroalgal assemblage between the survey time (SIMPER analysis); Abundant or maintained species are marked with a cross mark; Increased or decreased species are represented by their contribution (%); A (Abundant species) means that the average IV of the Chung’s survey and the present survey is above 5%; M (maintained species) is at least 5% in each of the IV in the Chung’s survey and the present survey; I (increased species) means that the increase in the IV is at least 1% and its contribution is at least 1%; D (decreased species) means that the decrease in the IV is at least 1% and its contribution is at least 1%.

	Site KS				Site PR				Site PC				Site AD			
	Macroalgal assemblage state															
	A	M	I	D	A	M	I	D	A	M	I	D	A	M	I	D
*Desmarestia antarctica*									X			7.9	X			9.1
*Desmarestia* complex	X			6.2	X	X	10.2		X		16.9		X	X		2.8
*Himantothallus grandifolius*	X	X			X			7.9	X	X		1.1	X	X	5.6	
*Phaeurus antarcticus*													X			
*Cystosphaera jacquinotii*					X			6.3				6.0				
*Ascoseira mirabilis*							4.2		X			2.6				
*Palmaria decipiens*	X	X		5.1			1.8					5.0	X			4.0
*Gigartina skottsbergii*			8.8				6.0					4.0	X	X	6.8	
*Iridaea cordata*					X	X		3.6				6.9	X			14.3
*Plocamium cartilagineum*	X	X			X	X	2.4		X		5.4		X		13.0	
*Curdiea racovitzae*								6.0					X		6.4	
*Georgiella confluens*	X			17.2				8.2								
*Myriogramme manginii*					X			13.4				5.0				7.4
*Picconiella plumosa*	X		24.4				3.3								8.3	
Cumulative contribution (%)	61.6				73.3				60.8				77.8			
Dissimilarity between survey times (%)	38.2				49.1				70.1				43.0

The pattern of change of the species causing the long-term change of the assemblage, the species that contributed to the dissimilarity of the Chung’s survey and the present survey communities derived from SIMPER analysis were 20 species; these species included *Desmarestia* complex and *Plocamium cartilagineum*, which were the most prominent species base on abundance during the past 30 years (Fig. 2, Supplementary Table S2). These two species were quantitatively (mean IV value) increased 2.8 and 1.6 times compared to the Chung’s survey. The abundance of *Himantothallus grandifolius* and *Palmaria decipiens* was very high at 10.4 in the Chung’s survey but decreased to 9.1 and 6.2 in the present survey, respectively (Fig. 2, Supplementary Table S2). Although the abundance of the two species present in high abundance in the assemblage decreased, the contribution to the assemblage change between survey times was less than 5.0%.

We tried to see how the species changed in the assemblage and how much the change contributed to the assemblage (Table 2). The changes in macroalgal assemblage in the survey site, i.e., within-site variation, were 38.2%, 49.1%, 70.1% and 43% in sites KS, PR, PC and AD, respectively (Table 2). Regardless of the survey time, there were 14 abundant species with an average abundance of more than 5%. The sum of the contribution of these species to the within-site variation was 60.8% − 77.8%, and explained the dissimilarity of the macroalgal assemblage between the survey times by at least 60%. *Desmarestia* complex, which made the largest contribution to between-survey difference (Supplementary Table S2), increased in sites PR and PC, and the contribution to changes in each site was 10.2% and 16.9%, respectively. Interestingly, the abundance of *Himantothallus grandifolius* was complementary to *Desmarestia* complex in site AD, where abundance of *Desmarestia* complex decreased, abundance of *H. grandifolius* increased, whereas site PR and PC showed the opposite pattern (Table 2, Fig. 2).

### Species composition and vertical distribution

**Figure 4:**
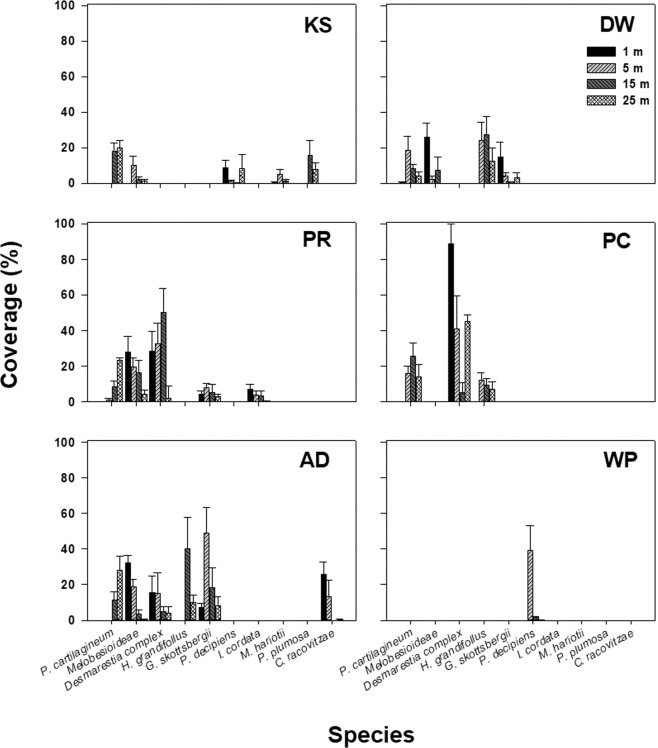
Composition of major algal species and vertical distribution at six sites in Maxwell Bay, King George Island; bar represents mean (+SE) percentage cover of species; Major species were selected based on the SIMPER contribution of each species (90% cut-off).

In the present survey, Daewang Rock site (site DW) was added to five sites analyzed in comparative analysis, thus the average coverage of each species, total algal cover of each site, and diversity were analyzed for six sites. *Desmarestia* complex*, Himantothallus grandifolius*, Melobesioideae (crustose coralline algae; CCA), *Palmaria decipiens, Gigartina skottsbergii, Plocamium cartilagineum,* and *Picconiella plumosa *appeared at least five of six sites, considered as common species in the study area (Fig. 4, Supplementary Table S3). Site DW, PR, PC and AD were high in macroalgal abundance with an average coverage of >50%. Particularly, site AD and PC showed 93.3% and 85.5% in macroalgal coverage, respectively, which were highest among the sites.

**Figure 5:**
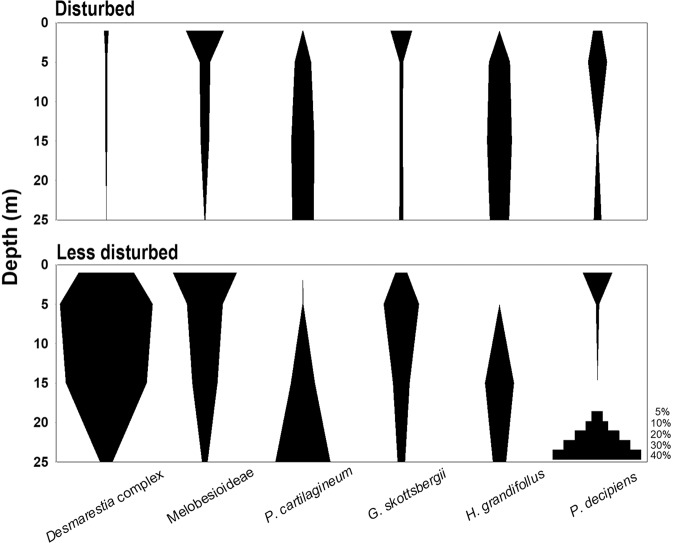
Vertical distribution of major species classified by habitat type (Disturbed by ice: site KS, DW, WP, Less disturbed by ice: site PR, PC, AD); the width of the graph indicates the coverage.

The distribution of seaweed species in each site and by depth derived from SIMPER analysis was as follows. *Desmarestia* complex, the most dominant species of the present survey, showed the highest coverage at 5 m depth and appeared most frequently at 5–15 m depth (Figs. 4 and 5). This appeared mainly in sites PR, PC, and AD outside Marian Cove (less disturbed site) and showed high site variability. Especially in site PC, the coverage of *Desmarestia* complex was about 90% at the depth of 5 m (Fig. 4), which resulted in low species diversity (Supplementary Table S3). *Plocamium cartilagineum* exhibited the highest coverage at 25 m depth, most frequently at 15–25 m depth (Fig. 5). *Himantothallus grandifolius* was mainly present at 15 m on all sites except site WP and was the most dominant species in site DW (Fig. 5, Supplementary Table S3). The major species of site WP was only one species*, P. decipiens* and it was found mainly in the shallow zone (Fig. 4).

In the sites PR and AD located near the penguin rookery, the macroalgal coverage was over 80%, which was much higher than the inner bay sites (site KS, DW, WP) and the number of species was as high as 17 and 18 species. In this area, the coverage was 65% or more even excluding the CCA, Melobesioidea. The diversity derived from the Shannon index was 2.05 and 2.23 at sites PR and AD, respectively, which were highest among the survey sites (Supplementary Table S3). These results were observed in both the present survey and the Chung’s survey (Table 1).

## Discussion

In most of the sites, notable pattern in the macroalgal assemblage over the last 30 years was the complementary increase or decrease of the *Desmarestia* complex and *Himantothallus grandifolius* populations which have the highest biomass in the Antarctic region and dominate in the sublittoral zone^18,29,30^ (Table 2). The complementary change and abundance shift can be interpreted in terms of temperature and inter-specific competition. First, Schoenrock, *et al*.^18^ reported that *Desmarestia menziesii* population would increase if the temperature increase due to global warming persisted. Glacier retreat has intensified due to warming over the past 50 years and the average temperature of the atmosphere has increased by over 1 °C in Marian Cove and Potter Cove, KGI^8,25,26^ (Fig. 1). Therefore, the increase of *Desmarestia* populations in KGI can not rule out the effects of climate change warming. Second, Klöser, *et al*.^31^ suggests that *D. anceps* and *D. menziesii* have a competitive advantage over *H. grandifolius* under favorable conditions (e.g. less ice impact). The inter-specific competition between *H. grandifolius* and *Desmarestia* complex was determined by disturbance caused by iceberg and sea ice fraction. Assuming an increase in temperature due to climate change and an increase in iceberg (from glacier retreat), an increase in *H. grandifolius* can be expected in site KS, an ice-abraded zone located inside the Marian Cove. *Desmarestia* complexes are expected to increase in site PR, PC and AD, where icebergs stay relatively short before they leave the open ocean. Also, *Desmarestia* spp. and *H. grandifolius* functionally act as kelps in Antarctic region^18^, and variation in their abundance can be understood as an increase or decrease of canopy. That is, changes in canopy can affect the fitness of understory species by inter-specific interactions^10^. Excessive canopy in site PC may have a negative effect on understory macroalgal assemblage. At the same time, canopies provide clues to understand the increase of the filamentous and foliose red algae *Plocamium cartilagineum* and *Gigartina skottsbergii* in sites PR and AD (Table 2)^32,33^.

The sites near the penguin rookery (site PR and AD) consistently showed the highest biodiversity, indicating an Antarctic coastal biodiversity hot spot. The presence of penguin breeding colonies is a major source of nutrients in the terrestrial ecosystem of Antarctica, affecting the formation of land vegetation and zonation^34^. The long-term change of macroalgal vegetation carried out on the Stora Bornö Island in the inner Gullmar Fjord on the Swedish Skagerrak coast was associated with an increase in nutrient availability^35^. Seabird guano has been shown to have a positive effect on some species (fleshy crustose algae, *Prasiola* and *Ulva*) at lower guano concentration in the intertidal zone, but in most cases guano had no direct effects^36^. Although seabirds can fertilize costal benthic communities through pelagic-benthic coupling, it can not be directly influenced by benthic primary production^37^. Nevertheless, one interesting point is that in site PR, unlike other sites, a rich benthic invertebrate assemblage was observed (personal observation, Supplementary Fig. S1). In particular, sponge, bryozoan, and soft coral populations were developed at depths of 15 m or more, suggesting that the state of hyper nutrients originating from the penguin rookery positively affects the richness of benthic animals as well as seaweeds.

Antarctica is generally known to have relatively low seaweed diversity^29,38^. For example, temperate southern Australia recorded approximately 1,500 species of macroalgal species^32^, while 130 macroalgal species have been recorded in Antarctica^38–40^. This low level of species richness is attributed to infrequent sampling, difficulty of access, and logistic difficulties^41^. Also, the use of quadrat photo is difficult to identify understory species or cryptic species, and because Antarctica is a relatively unexplored region, macroalgae is still less taxonomically resolved. About 30 species of algal species were found in this study and 25–66 species of seaweed were reported in the previous studies conducted in KGI. Studies of taxonomic reports have shown relatively high species richness^41–43^, but are also closely related to relatively low latitudes compared to other regions of Antarctica. In case of ecological studies or a small number of survey sites, the level of species richness was similar to our study^44,45^.

30 years snapshot survey revealed that there were significant changes in the species composition (Fig. 2, Table 1). This shows that the assemblage in this area was changing very dynamically, but the heterogeneity or homogeneity between sites determined by various environmental factors did not seem to change. We chose ecologically diverse sites that could represent Maxwell Bay among the sites of the Chung’s survey, so the similarity of the macroalgal assemblage among the sites was inevitably low. The past and present hierarchical structures based on similarity are clearly distinguished from sites KS and WP inside of Marian cove (frequently disturbed by ice) and sites PR, PC and AD exposed to open ocean (less disturbed by ice). The changes in the benthic community in Potter Cove, KGI showed that the community in the same site showed a statistically significant difference in four to six years^46^. Sahade, *et al*.^46^ reported that sudden shifts in the benthic community occurred due to sedimentation caused by glacier retreat. In the Antarctic area, the community is determined by various environmental factors such as iceberg scouring, sedimentation from glacier retreat, ice cover and light, but it is very difficult to interpret community dynamics due to the complexity between these environmental conditions. The macroalgal assemblage, we report here, which was analyzed over 30 years, maintained a hierarchical structure based on the similarity between the five sites despite the dynamic change of the assemblage. Unfortunately, our long-term snapshot study does not include the causes of macroalgal assemblage changes (eg. temperature, disturbance, sediments) and the patterns of change (eg. linear, sudden, threshold). Despite the apparent change in assemblage, maintaining the hierarchy could be considered an example of an alternative stable state^47–49^ in Antarctica. Therefore, it is necessary to confirm whether the variability of macroalgal assemblage and the stability of hierarchical structure are maintained regardless of the time interval, through short term monitoring in the future.

Finally, our study has great significance in trying to analyze the long-term change of algal assemblage at the same sites over an interval of about 30 years in the Antarctic region. In Gullmar Fjord on the Swedish Skagerrak coast, macroalgal vegetation was discovered at the same site as it was 57 years ago. Its continuing presence was interpreted as related to the nutrient dynamics of Baltic Sea and Kattegat water as an exceptional example of analyzing historical assemblage change through comparison with existing research results^35^. In Arctic fjords along the coast of Svalbard, where sea water temperature increases and sea ice area decreases, the macroalgal coverage increased 5 to 8 times over 30 years^20^. Warming in the Arctic region increases turbidity under the influence of sediments in sea ice and decreases salinity under the influence of melting ice. Those increase of turbidity and decrease of salinity have a negative effect on kelp recruitment, growth, and survival. As a result, the positive effects of warming and increased light can be offset^10^. In the sub-Antarctic Prince Edward Islands, a comparison of an epibenthic assemblage over a 25-year time difference confirmed the increase in polychaete *Lanice marionensis* (a suspension feeder); the cause was interpreted as a change in the local primary productivity due to climate change, that was, the change in the flux of food^50^. In the Arctic region, the biomass of *Laminaria digitata* was reported to have increased about 8.2 times compared to 16 years ago^21^. Thus, recent studies on the long-term change of cold water communities have been reported with regard to climate change or warming but there are not many cases in the Antarctic region. Mystikou, *et al*.^22^ compared the similarity and shared species among sites several kilometers to several hundred km away from the islands near the south-western Antarctic Peninsula. Unfortunately, this study was not a site based comparative survey because the sites, surveyed over a 35-year time period, were more than 350 km apart. Therefore, our study is very meaningful in that it is a site based comparative survey in Antarctica.

In conclusion, we compared the macroalgal assemblages of about 30 years ago and later in Maxwell Bay, KGI to initiate a long-term research in the Antarctic region. We observed that large brown algae populations dominant in the Antarctic regions shifted from the *Himantothallus grandifolius* to the *Desmarestia* complex. Previous studies have shown that this population shift is strongly suspected to have resulted from a competitive replacement of *H. grandifolius* by *Desmarestia* complex. If global warming intensifies in the future, the *Desmarestia* complex population will expand directly due to the increase in water temperature. As seen from the site nearest the glacier (site WP), a change to a simple assemblage dominated by r-type algae such as *Palmaria decipiens* or *Acanthococcus antarcticus* can be expected. In addition, considerable changes were observed in the species composition of past and present macroalgal communities, that is, within-site variation was very high. We expected that the spatial variation would also be very large if this dynamic transition of macroalgal communities was common in the KGI coast, but between site heterogeneity did not appear to be significant. This suggests that climate change such as global warming leads to drastic change of macroalgal assemblage in local micro scale (- up to 1 km^2^; within sites) but it shows a gradual change in regional meso scale (1–10,000 km^2^; among sites) at Maxwell Bay in KGI. Also we hope that this study will serve as another important basis for the interpretation of future macroalgal assemblage changes in the Antarctic region due to climate change.

## Materials and methods

### Study area

This study was conducted in Maxwell Bay, King George Island (KGI), the largest island of the South Shetland Islands, about 120 km far from the Antarctic Peninsula. KGI is about 95 km long and 25 km wide with a land area of 1,150 km^2^. More than 90% of the land area is covered with permanent glaciers, and ice-free land areas are limited to the coast. Coastal areas connected to exposed land areas are regularly frozen from late July to mid-September of the austral winter, and after late October, the sea ice begins to crack and floating ice starts to cover the coastal zone. This study was carried out at four sites (KS, DW, PR, PC) around Barton Peninsula, one site (WP) around Weaver Peninsula and one site (AD) near Ardely Island (Fig. 1, Table 3). Site KS was located in front of the pier of King Sejong Station, so an anthropogenic effect is expected. Sites PR and AD were located in the vicinity of the penguin rookery, where a large amount of organic nutrients are introduced from the land. Sites KS, DW and WP (located in Marian Cove) were influenced by the sediments that flowed along with melted snow water and turbid plume from glacial retreat^51^. Predominant winds direction (NW-W) increase the residence time of the ice within the cove, and strong wave generated by strong wind intensity forms the ice-abraded zone. These hydrodynamics of ice in Marian Cove are expected to be similar to the hydrodynamic pattern shown in Potter Cove^52^. On the other hand, sites PR, PC and AD are relatively less affected by disturbance caused by ice. Especially, the terrain near site WP formed a steep slope, and large and small pebbles rolled down from the slope and piled up on the shore and underwater. Here, mud was piled up on the bottom and the development of seaweed assemblage was poor.

**Table 3:** List of sampling sites and the characteristics in the Chung’s survey and the present survey; The Chung’s survey was conducted from 1988 to 1993 and the present survey was conducted from 2016 to 2018.

Site	Geographical location	Longitude and latitude	Sampling time		Substratum	Slope	Remarks
			Chung’s survey	Present survey			
KS	pier of King Sejong Station	62.222° S, 58.788° W	X	X	rock, boulder with mud (>5 m: pebble)	steep	Frequently disturbed by ice, post anthropogenic effect
DW	Daewang Rock	62.219° S, 58.776° W		X	rock, boulder with mud (>5 m: stepped rock with no mud)	steep	Frequently disturbed by ice, glacier retreat and melt water effect
PR	an islet off penguin rookery	62.240° S, 58.777° W	X	X	rock with sand	steep	Less disturbed by ice, near the penguin rookery, nutrient enrichment
PC	an islet in the entrance of Potter Cove	62.237° S, 58.714° W	X	X	rock with sand	steep	Less disturbed by ice
AD	an islet off Ardley Island	62.218° S, 58.914° W	X	X	rock	steep	Less disturbed by ice, near the penguin rookery, nutrient enrichment
WP	a southern shore of Weaver Peninsula	62.206° S, 58.769° W	X	X	pebble, boulder with mud	steep	High sedimentation from lands (melting ice)

### Sampling for the present survey and the Chung’s data

The field survey was conducted at the six sites in Maxwell Bay, KGI, during the austral summer 2016–18. The sampling areas were all located outside of the Antarctic Specially Protected Area (ASPA no. 171, site PR; ASPA no. 150, site AD), and all surveys were performed with non-destructive sampling to minimize disturbance to the ecosystem. Algal assemblage of subtidal habitats was investigated by SCUBA diving with underwater camera and video. Diving survey was performed at four depths (1 m, 5 m, 15 m, and 25 m) and were conducted along a virtual vertical line. Five 50 × 50 cm (0.25 m^2^) quadrats were randomly located at each depth and photographed. Quadrats, within the same depth, were placed >2 m apart from each other were horizontally placed. The underwater video device was used to record the substrate configuration and the surrounding environment while diving down to the depth of 25 m. We counted the coverage (percentage) of each macroalgal species using virtual grids on the photo. Algal identification was done to the lowest possible taxonomic level. Exceptionally, crustose coralline algae were classified at subfamily level.

Unfortunately, there is no raw data available in the Chung’s survey, except for the published data. Those data was coverage and/or biomass and then they converted them to IV (Supplementary Table S4). Along the transect line, macroalgal assemblage data in quadrat (1 m × 1 m) were collected at different depth intervals (2 m interval between 0–10 m and 5 m interval between 10–30 m). Also, the data were presented as the average value of each site and there was no depth profile (more details in Chung’s literatures^27,28,53^).

### Data analysis for comparing surveys

All the data collected in the present survey were converted to the same format as the data published in the Chung’s survey, to achieve direct comparison between the Chung’s survey and the present survey. Major species were selected to adequately express the differences in communities in both surveys. Major species were defined by the following method. The similarity of percentage (SIMPER) analysis^54^ was conducted between the surveys at the average abundance data of each site. Eight species with the contribution of more than 5% to the difference between the two surveys were defined as the major species (for Fig. 3 and Supplementary Table S2). The composition of assemblage is presented by calculating the cumulative contribution or by sorting the contribution of all species in ascending order^55–59^. To understand the variation of the assemblage (for Table 2), we defined the species with an average abundance of 5% or more in the Chung’s survey and the present survey as abundant species. Despite the 30-years time lag of the two surveys, such species that showed more than 5% abundance in the Chung's survey and the present survey each were expressed as maintained species because the macroalgal population remained stable. Moreover, we observed the patterns of variation of the communities by examining how much the increase or decrease of the population contributed to the within-site variation. The biomass and coverage of species in site WP were very low compared to other sites, so site WP was excluded from the comparison, preventing a distortion in major species calculation and their contribution. Cluster analysis was performed to investigate the spatial variation between sites for each survey. For this analysis, Bray Curtis similarity between sites was calculated, then a resemblance matrix was created and a hierarchical cluster analysis was performed to create a dendrogram (for Fig. 3 and Supplementary Table S1). The algorithm for calculating the distance between clusters was group average. Prior to SIMPER and cluster analysis, all data were subjected to a pre-treatment procedure through standardized and square root transformation^54^. Shannon-Wiener diversity index was calculated to compare the diversity of the algal assemblage between sites^60^.

### Macroalgal assemblage and vertical distribution in the present survey

We attempted to identify the distribution of major species by depth at each site using the extracted coverage-based data in the present survey. Major species were selected for each site to describe the vertical distribution of macroalgal assemblage (for Figs. 4 and 5). Major species were selected based on the contribution of each species (90% cut-off) to similarity between the site through SIMPER analysis. Cluster analysis, diversity index and the pre-treatment process of data for assemblage analysis were done as described above. The SIMPER, cluster analysis, and diversity index analysis applied to this study were performed using PRIMER 6 software (PRIMER-E, Ltd., UK).

## Supplementary information

Supplementary information.

Supplementary information2.

Supplementary information3.

Supplementary information4.

Supplementary information5.
