# Supplementary material for: 30 years revisit survey for long-term changes in the Antarctic subtidal algal assemblage
Source: Sci Rep. 2020 May 21;10:8481. doi: 10.1038/s41598-020-65039-4 (PMC7242392; doi:10.1038/s41598-020-65039-4)
Supplement: Supplementary file 1 — Supplementary information. [file 41598_2020_65039_MOESM1_ESM.pdf]

Supplementary information

30 years revisit survey for long-term changes in the Antarctic subtidal algal assemblage

Young Wook Ko[1,2], Han-Gu Choi[1], Dong Seok Lee[2], Jeong Ha Kim[2*]

[1] Department of Polar Life Sciences, Korea Polar Research Institute, Incheon 21990, Republic of Korea

[2] Department of Biological Sciences, Sungkyunkwan University, Suwon 16419, Republic of Korea

*To whom correspondence should be addressed

Telephone: +82-31-290-7009

Fax: +82-31-290-7015

E-mail: jhkbio@skku.edu

Supplementary Tables

Supplementary Table S1. Similarity matrix (Bray-curtis similarity index) between sites in each survey of the macroalgal assemblage base on importance value (IV), and dissimilarity (SIMPER) between the Chung's survey and the present survey at each site

	Similarity matrix										Dissimilarity
	Chung's survey					Present survey					
	KS	PR	PC	AD	WP	KS	PR	PC	AD	WP	
KS											38.18
PR	49.62					45.74					49.11
PC	24.12	55.42				45.88	62.19				70.06
AD	36.20	59.40	50.55			51.91	51.82	57.03			42.95
WP	41.67	25.26	28.76	33.63		33.04	27.88	20.78	20.43		53.36

Supplementary Table S2. Similarity Percentage (SIMPER) analysis of dissimilarity between the Chung's survey and the present survey

Species	Mean importance value		Contribution (%)	
	Chung's survey	Present survey		Cumulative
Desmarestia complex*	10.7	30.3	8.9	8.9
Plocamium cartilagineum	12.1	19.6	7.7	16.6
Iridaea cordata	9.2	1.5	6.7	23.3
Myriogramme manginii	8.7	0.3	6.7	30.0
Picconiella plumosa	0.3	7.3	6.1	36.1
Georgiella confluens	5.9	0.1	5.5	41.5
Desmarestia antarctica	7.7	0.6	5.4	47.0
Gigartina skottsbergii	4.8	9.8	5.3	52.3
Palmaria decipiens	10.4	6.2	4.9	57.2
Cystosphaera jacquinotii	5.9	1.0	4.8	62.0
Acanthococcus antarcticus	0.0	3.4	4.6	66.7
Curdiea racovitzae	1.8	3.3	4.0	70.7
Ascoseira mirabilis	2.8	2.6	4.0	74.7
Phaeurus antarcticus	2.9	0.6	3.4	78.1
Monostroma hariotii	1.3	1.6	2.8	80.9
Himantothallus grandifolius	10.4	9.1	2.8	83.7
Trematocarpus antarcticus	0.9	0.2	2.0	85.7
Pantoneura plocamioides	0.9	0.0	1.9	87.6
Ulva bulbosa	1.2	0.0	1.5	89.0
Neuroglossum ligulatum	0.0	1.0	1.5	90.5

* mixed with *D. anceps* and *D. menziesii* (mainly composed of *D. menziesii*)

Supplementary Table S3. Composition of algal species and mean coverage at six sites in Maxwell Bay, King George Island during the present survey

Taxon	Site					
	KS	DW	PR	PC	AD	WP
**Chlorophyta**						
Monostroma hariotii	1.89±0.88			0.07±0.07		0.60±0.31
Ulothrix australis	0.57±0.57					
**Phaeophyta**						
Petroderma maculiforme			0.08±0.08			
Adenocystis utricularis		0.25±0.25				
Halopteris obovata					0.30±0.22	
Desmarestia antarctica			0.55±0.55	0.27±0.27	1.00±0.55	
Desmarestia complex[1]	1.20±0.67	0.80±0.80	28.63±5.73	46.73±11.08	11.75±3.92	
Himantothallus grandifolius	3.09±1.79	16.00±4.26	0.68±0.41	7.13±2.28	12.65±5.58	
Phaeurus antarcticus				0.20±0.20	1.75±0.58	
Cystosphaera jacquinotii			2.60±2.50			
Ascoseira mirabilis	0.20±0.20	3.73±2.11	4.00±1.84	3.00±3.00		
**Rhodophyta**						
Melobesioideae[2]	3.94±1.66	8.95±3.09	16.93±3.37	6.60±2.89	13.75±3.28	
Ballia callitricha		0.35±0.35				
Delisea pulchra					0.15±0.15	
Palmaria decipiens	4.11±2.36		4.00±2.34		3.85±2.19	13.60±6.52
Callophyllis linguata					0.65±0.65	
Trematocarpus antarcticus					1.35±0.94	
Acanthococcus antarcticus			4.30±2.65	4.80±3.25	0.95±0.65	1.60±1.60
Gigartina skottsbergii	1.26±0.60	5.85±2.32	5.05±1.37	0.67±0.37	20.75±5.84	
Iridaea cordata	0.06±0.04	7.28±2.84	3.70±1.10			
Phyllophora ahnfeltioides		0.08±0.08				
Plocamium cartilagineum	10.8±2.38	8.00±2.34	8.13±2.37	13.93±3.83	9.85±3.40	0.40±0.34
Curdiea racovitzae				0.53±0.41	9.80±3.62	
Georgiella confluens	0.11±0.09		0.03±0.03			
Myriogramme manginii	0.03±0.03	0.15±0.15		0.20±0.20	0.70±0.42	
Neuroglossum ligulatum			2.65±1.23			
Pantoneura plocamioides			0.03±0.03			
Phycodrys antarctica			0.13±0.13			
Paraglossum lancifolium			0.25±0.16			
Picconiella plumosa	6.86±2.79	0.10±0.08	0.75±0.43	0.47±0.47	3.85±1.91	
Unidentified species[3]				0.87±0.87	0.15±0.15	
Total species	13	13	18	14	17	4
Total coverage (%)	34.1	52.5	82.5	85.5	93.3	16.2

Shannon index	1.94	1.89	2.05	1.51	2.23	0.59

[1] mixed with *D. anceps* and *D. menziesii* (mainly composed of *D. menziesii*)

[2] Melobesioideae contained several species of crustose coralline algae and pooled as a taxa because of difficulty in collecting and identifying

[3] not identifiable on the photograph

Supplementary Table S4. The comparisons of method for the Chung's survey and the present survey

	Chung's survey	Present survey
Depth	Transect interval*	1, 5, 15, 25m
Replication number	-**	5
Quadrat size	1 m x 1 m	0.5 m x 0.5 m
Data parameter	Coverage or Biomass	Coverage
IV***	RC	RC
Strength	Surveyed at same sites and directly compared by site<br>1st revisit algal assemblage survey in Antarctica<br>Present detailed spatial assemblage in Maxwell Bay.	
Weakness	Not a perfectly matched parameters<br>Different quadrat size and sampling depth	

* 2m interval between 0-10m and 5m interval between 10-30m, no exact depth profiles
** not indentified
*** IV = (RC+RB)/2; IV (Importance Value); RC (Relative Coverage); RB (Relative Biomass)

Supplementary Figure

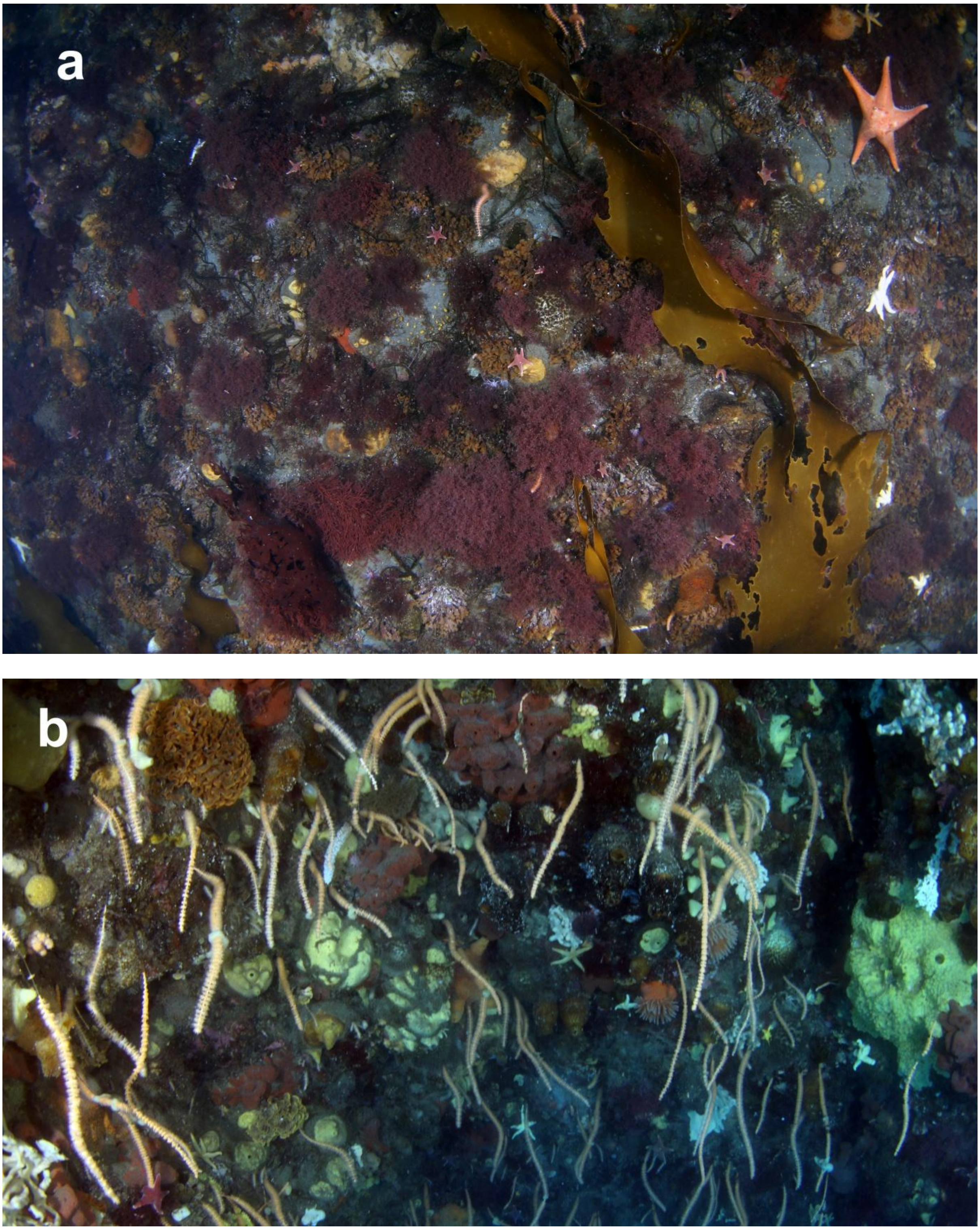

Supplementary Figure S1. a; *Himantothallus grandifolius* and *Plocamium cartilagineum* with diverse benthic invertebrates at 25 m depth in site PR, **b**; diverse benthic invertebrates (soft corals, sponges, sea anemones, sea stars, tunicates etc.) at 30 m depth in site PR
